# Profiling of Discrete Gynecological Cancers Reveals Novel Transcriptional Modules and Common Features Shared by Other Cancer Types and Embryonic Stem Cells

**DOI:** 10.1371/journal.pone.0142229

**Published:** 2015-11-11

**Authors:** Kalliopi I. Pappa, Alexander Polyzos, Jasmine Jacob-Hirsch, Ninette Amariglio, George D. Vlachos, Dimitrios Loutradis, Nicholas P. Anagnou

**Affiliations:** 1 First Department of Obstetrics and Gynecology, University of Athens School of Medicine, Alexandra Hospital, Athens, Greece; 2 Laboratory of Cell and Gene Therapy, Biomedical Research Foundation of the Academy of Athens, Athens, Greece; 3 Basic Research Centre, Biomedical Research Foundation of the Academy of Athens, Athens, Greece; 4 Cancer Research Center, The Chaim Sheba Medical Center, Tel-Hashomer and Sackler School of Medicine, Tel Aviv University, Tel Aviv, Israel; 5 Laboratory of Biology, University of Athens School of Medicine, Athens, Greece; University of Nebraska Medical Center, UNITED STATES

## Abstract

*Studies* on individual types of gynecological cancers (GCs), utilizing novel expression technologies, have revealed specific pathogenetic patterns and gene markers for cervical (CC), endometrial (EC) and vulvar cancer (VC). Although the clinical phenotypes of the three types of gynecological cancers are discrete, the fact they originate from a common embryological origin, has led to the hypothesis that they might share common features reflecting regression to early embryogenesis. To address this question, we performed a comprehensive comparative analysis of their profiles. Our data identified both common features (pathways and networks) and novel distinct modules controlling the same deregulated biological processes in all three types. Specifically, four novel transcriptional modules were discovered regulating cell cycle and apoptosis. Integration and comparison of our data with other databases, led to the identification of common features among cancer types, embryonic stem (ES) cells and the newly discovered cell population of squamocolumnar (SC) junction of the cervix, considered to host the early cancer events. Conclusively, these data lead us to propose the presence of common features among gynecological cancers, other types of cancers, ES cells and the pre-malignant SC junction cells, where the novel E2F/NFY and MAX/CEBP modules play an important role for the pathogenesis of gynecological carcinomas.

## Introduction

Gynecological cancers represent more than 10% of the cancers of the female population. Early diagnosis of the malignancy offers higher cure and survival rates and a better quality of life, in contrast to diagnosis at advanced stages that leads to radical operations and carries a higher percentage of morbidity and mortality. Specifically, the major types of gynecological cancer include cervical cancer (CC), endometrial cancer (EC) and vulvar cancer (VC), exhibiting an overall incidence of 7.4, 25.5 and 2.5 per 100,000 women-years, respectively [1].

The clinical phenotypes, the degree of the causal relation of HPV infection, along with the prognostic factors, such as stage, histology, histological grade, age at diagnosis and race [1], and the pathogenetic mechanisms involved for each of these three types, seem to be discrete and specific. However, all three types originate from an almost common embryological origin, such as the paramesonephric (Müllerian) ducts arising from the mesoderm during the eighth week of development via a process referred as Müllerian organogenesis and lying in the same anatomical region [2]. This observation, has led to a hypothesis that the major molecular and biochemical events and the ensuing aberrant pathways occurring during carcinogenesis in the three types, might share common features that reflect aspects of regression to early development and embryogenesis. Indeed, such findings have been recently documented in cancers other than gynecological ones [3].

For that reason,–as a first step–novel gene expression profiling technologies are currently applied by several groups [4,5] and ours [6] in gynecological cancers to reveal specific pathogenetic patterns in gene expression programs between healthy and cancer cells. Recently, further studies using next generation sequencing technologies, microarrays and proteomics, have focused on the genome mutation rate, the profiling of genome expression, and the proteome pattern of the individual gynecological cancers, i.e. vulvar [7], cervical [8] and endometrial [9] cancers, and have eventually composed a rather complete profile for each gynecological cancer. These approaches have led further to a novel grouping system of the tumor types [3,9–11].

Intriguingly, although common features emerge from studies among cancers of different origin regarding mutation rates, expression profiles or DNA methylation patterns [3,11], to our knowledge very few studies [12–14] have illustrated common biological functions or molecular mechanisms across different cancer types. Recently, a few comparative studies [15,16] utilizing the new profiling technologies in combination with the available bioinformatic tools [17] for the construction of biological networks, have identified common system–level properties among different cancer types [3,12–15].

Therefore, in view of the above hypothesis on the common origin of the gynecological cancers exhibiting common features, and the paucity of relevant data in the field, in this study we aimed to get further insights into these issues by performing a systematic and comprehensive molecular comparative characterization of endometrial, cervical, and vulvar cancer types. Our data identified both common and novel distinct modules controlling the same deregulated biological processes in all three types. The comparison of our results with other gene signature databases [18,19], led to the identification of common features among various cancer types and gynecological cancers. Consequently, we searched for candidate common deregulated genes in the three gynecological cancer types by comparing our findings with previously identified potential biomarkers of earlier studies, building a more robust gene signature for each type of cancer. Though we found a very small overlap in the gene signatures of the same cancer type in different studies, affected biological processes and deregulated molecular mechanisms were the same in most of the cases. Thus, the present study suggests that even if specific genes can act as drivers or biomarkers, cancer cells maintain the capacity to arrive to the same end-stage, by activating and repressing different gene nodal points of a molecular mechanism or a pathway.

## Results

### Common features of endometrial, cervical and vulvar carcinoma

A total of 35 samples derived from patients with gynecological cancers of different histology at different stages and healthy controls (S1 Table) were analyzed. Specifically, 18 cancer samples (5 cervical, 7 endometrial and 6 vulvar) and 17 normal samples (5 cervical, 5 endometrial and 7 vulvar) were hybridized on Affymetrix platform as previously described [6]. Profiling of gynecological cancers revealed 1406 (762 upregulated and 644 downregulated), 1740 (733 upregulated and 1007 downregulated) and 1679 (448 upregulated, 1231 downregulated) differentially expressed genes (DEGs) in cervical, endometrial and vulvar cancer, respectively (S2 Table). Principal component analysis (PCA) on differentially expressed genes, discriminated normal from cancer samples of the same tissue type (S1 Fig), while cancer or normal samples from endometrium and cervix were closer to each other, suggesting a common embryonic origin.

**Fig 1:**
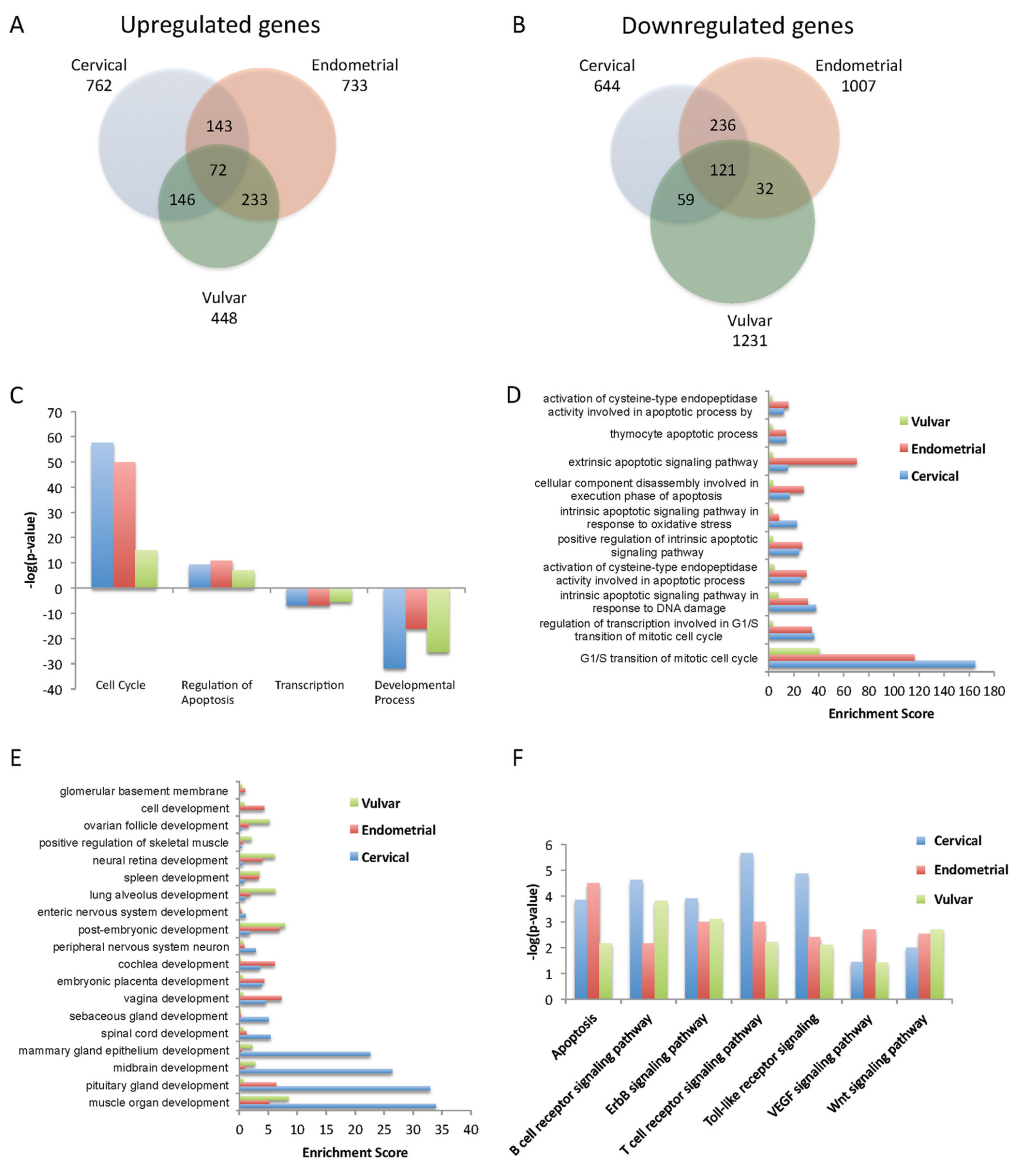
Comparison between differentially expressed genes in the three gynecological cancers and characterization of common biological processes and pathways. A. Venn diagrams comparing upregulated. B. Downregulated differentially expressed genes in cervical, endometrial and vulvar cancers vs. their corresponding normal samples. C. Top biological processes. D. Comparison of the enrichment of cell cycle and apoptosis related categories in the three gynecological cancers as identified by Comparative GO [17] in the upregulated gene lists and in the E. downregulated gene lists. F. Pathways deregulated in all gynecological cancers are depicted in bar graphs.

Comparison of the three gynecological cancers vs. their corresponding normal samples, revealed a 15–40% overlap between each type of cancer (Fig 1A and 1B) with only 193 common differentially expressed genes (72 upregulated and 121 downregulated). Despite the fact that overlaps among cervical, endometrial and vulvar gene signatures were small, gene ontology analysis showed increased overlap among them regarding their biological processes. Clear separation between biological functions mediated by upregulated and downregulated genes was noted. Cell cycle, apoptosis and regulation of apoptosis were among the categories enriched in the upregulated population of all three gynecological cancers of the study. Downregulated genes were found to be involved in transcription and various developmental categories such as muscle, skeletal and blood development (Fig 1C). Focusing on the common deregulated biological processes and comparing the folds of change of those genes, we noticed that cell cycle and apoptosis-related biological processes, were more affected in cervical and endometrial cancer than in vulvar cancer cells (Fig 1D and S3 Table). Developmental categories were more diverse (Fig 1E) and we were able to identify developmentally related processes mostly enriched in cervical (muscle organ development) or vulvar and endometrial cancer cells (embryonic placenta development). Human papillomavirus (HPV) represents the main factor for cervical cancer [20], and as expected, the biological process of ‘response to virus’ was enriched only in the upregulated gene population of patients with cervical cancer, with *Mx1*, interferon regulating factors 7 and 9 (*Irf7*, *Irf9*), and interferon-stimulated genes 15 and 20 (*Isg15*, *Isg20*), exhibiting high expression levels and thus confirming the activation of antiviral response-related genes in HPV-infected cells (S2A Fig).

### Identification of common pathways and networks in the development of gynecological cancers

Pathway enrichment analysis was performed utilizing Expander 5.2 [21,22] and DAVID knowledgebase 6.7 [23,24]. Information from KEGG, REACTOME, BIOCARTA and PANTHER pathway databases was retrieved and used for the identification of differentially expressed genes involved in the upregulation or downregulation of known pathways. Utilization of different databases provided us the advantage to annotate our generated gynecological cancer signatures in pathways that were represented only in one database. This annotation revealed several novel features of gynecological cancer signaling pathways known to be involved in cancer formation, development and immune response, which were enriched in cervical, endometrial and vulvar cancer samples (Fig 1F). Wnt, ErbB, Vegf pathways were mainly enriched in the downregulated gene population. Wnt pathway, which is known to regulate transcription activity and cause aberrant cell division and migration associated with cancer formation [25–27], was found indeed deregulated as documented by the downregulation of *Wnt2*, *Nfatc1*, *and Nfatc4* genes. It is well established that ErbB pathway receptor misregulation and excessive signaling is associated with the development of cancer. In our study, although *ErbB*-2 was upregulated in endometrial cancer, *ErbB*-2 and *ErbB*-3 were downregulated in patients with vulvar cancer. B cell and T cell receptor signaling pathways, which are involved in the immune response system, were also affected. Key gene components such as *Nras* (upregulated in all gynecological cancers), *Pik3ca* (upregulated in cervical cancer, downregulated in endometrial cancer), and *Jun*, *Akt3* (downregulated in endometrial and vulvar cancer), which are also involved in known cancer- related pathways, showed variable expression in all three gynecological cancers. In this study, we observed that *Arid1a* exhibits a statistically significant downregulation both in endometrial and vulvar cancer patients, but not in cervical cancer patients (S2B Fig). This finding suggests common aberrantly operating mechanisms controlling alterations in PI3K-Akt and TP53 signaling pathways leading to tumor formation in both cancer types [28].

In concordance with the previous results from gene ontology analysis, most of the upregulated and downregulated genes formed networks, in which the main effect of interacting genes in all gynecological cancer types is depicted in Cell Cycle and Immunological Disease for upregulated interacting genes, and in Cellular Growth and Proliferation, Cellular Development, Cell Death and Survival and other metabolic and morphogenesis-related categories for downregulated interacting genes (S3A and S3B Fig). Similar networks were formed when we analyzed the common regulated genes in all gynecological cancers. The Cell Cycle-related network exhibited the second highest score, while the Cancer and Cell Death-related network displayed the third highest score (S4A and S4B Fig).

### Cancer-related pathways and gene signatures

**Fig 2:**
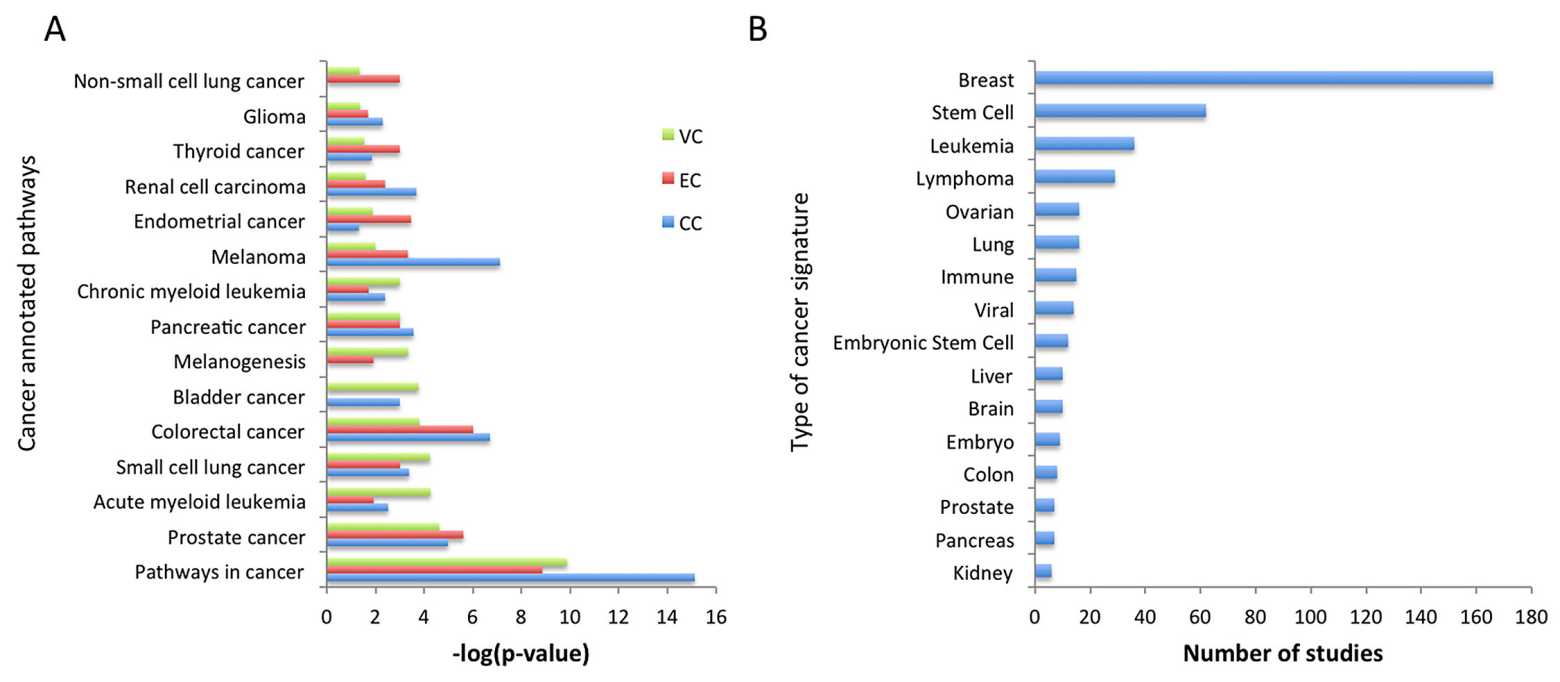
Cancer-related pathways and signatures enriched in gynecological cancers. A. Bar graphs showing the significance of overlap between the three gynecological cancers in this study with other known types of cancers and their annotated pathways. B. Oncogenic gene signatures from MSigDB 4.0 [14] and GeneSigDB 4.0 [15].

Based on the above findings, we then investigated for the presence of common pathway signatures in other types of cancers. To this end, employing data from annotated pathway databases, we noticed that breast, pancreatic, prostate, and colorectal cancers correlated with cervical, endometrial and vulvar cancer pathways (Fig 2A). This result was strengthened when our signatures were compared to those annotated in MSigDB 4.0 [18] and GeneSigDB 4.0 [19] databases. In concordance with the pathway results, most of the signatures that overlapped with our study, i.e. with more than 5 genes in common and *p* < 0.01, were derived from breast, lung and prostate cancer, which were among the top ones (Fig 2B). Notably, overlap between our differentially expressed genes was found with many stem cell signatures, which were the second, most frequently correlated gene signature after breast cancer. Viral and immune response-related signatures were also enriched in all the differentially expressed genes for all three types of gynecological cancer of the study. Search in MSigDB 4.0 [18] database for oncogenic signatures disclosed a significant overlap between all three gynecological cancers with lung (5 signatures), breast (9 signatures) and prostate cancer (5 signatures), as shown in S2 Table. All these data highlight the fact that although there are numerous genes that are deregulated in each cancer type, only a small percentage of common ones can be found in multiple types of cancer. In most cases, overlap does not exceed 20 to 30 genes. A summarization of all those genes that are differentially expressed in more than five studies annotated in GSEA 2.0.14 [18], created a gene list, which included genes with higher probability to occur or to be involved in the development of tumors. A total of 81 genes was found through GSEA 2.0.14 [18] to be represented as a gene signature in more than five studies, with *Fos* and *Ccnd2* involved in Cell Cycle process, *Anxa1*, *Birc3*, *Socs2*, *Gch1*, and *Chst11* involved in Regulation of Apoptosis, and various transcription regulators such as *Id2*, *Klf4*, *Bcl3*, *Satb1*, *Egr1*, and *Fos* which are enriched in multiple tissue malignancies. Thus, our approach can provide the opportunity for future additional comparisons with other known gene signatures and scoring genes, based on the frequency of occurrence in various diseases or cancer-related categories (Table 1).

**Table 1:** Genes upregulated in various gynecological studies. List of genes differentially expressed in any of our three gynecological cancer types that overlapped with more than five oncogenic signatures, as documented from the GSEA database, forming a robust oncogenic signature for GCs and other types of cancer.

A2M	FERMT2	NYNRIN
ABLIM1	FGF9	PCDH9
ACOX2	FOS	PDCD4
AKAP12	FZD2	PDGFRL
AQP3	GAS1	PDLIM3
ATF3	GAS7	PEG3
AXL	GNG11	PID1
BCL2	GOLGA8A	PLSCR4
BEX4	GPR137B	PLXND1
C1orf115	GPX3	PPP1R3C
CAPRIN2	HBP1	PRIM1
CCND2	ID2	PTBP2
CDC42EP3	ID4	QPRT
CLIP3	IL1R1	RASL12
CRABP2	IL33	RGS2
CRIM1	IRS1	RRM2
CTSH	IRS2	SASH1
CYFIP2	ISL1	SATB1
CYP1B1	ITGBL1	SCARA3
DAB2	KCTD12	SEPP1
DLC1	KIT	SLIT3
DNAJC12	KLF4	SMARCD3
EFEMP1	L1CAM	SOCS2
EFHD1	LRIG1	SPRY2
EGR3	MATN2	TSPAN7
EMP1	METTL7A	TXNIP
EZH2	NUDT11	UBE2C

### Correlation of cervical cancer differentially expressed genes with the newly identified cell-population from the cervical squamocolumnar junction

**Fig 3:**
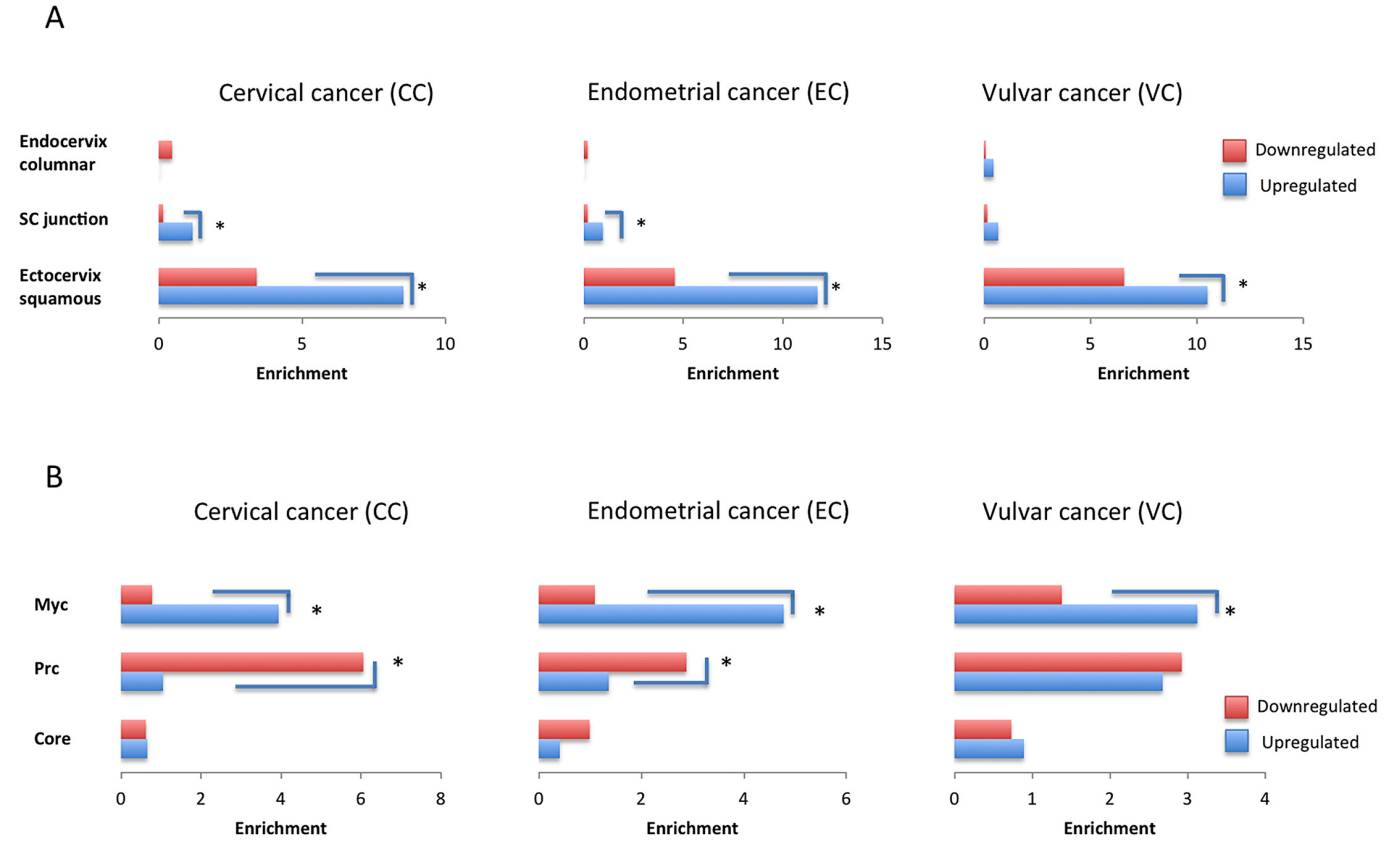
Endocervix columnar, squamocolumnar and ectocervix squamous gene signatures and ES enrichment in cervical, endometrial and vulvar cancer patients. A. Enrichment of differentially expressed genes from our gene signatures of cervical, endometrial and vulvar cancer with endocervix columnar, squamocolumnar junction (SC) and ectocervix squamous gene signatures. For significant differences with *p* < 0.05, the asterisk was used for annotation. B. Enrichment of differentially expressed genes from our gene signatures from cervical, endometrial and vulvar cancer with Myc, Prc and Core modules, identified to play a key role in the formation and establishment of pluripotency in embryonic stem cells [33]. For significant differences with *p* < 0.05, the asterisk was used for annotation.

Recently it was suggested that cervical cancer originates from a small number of a discrete population of cuboidal epithelial cells located at the squamocolumnar (SC) junction at the ectoendocervical area of the cervix [25]. These data provided us the opportunity to test whether this unique population with a potential for malignancy shares common features with established types of gynecological cancers. Overlap between our upregulated differentially expressed genes in endometrial and cervical cancer patients and upregulated genes in the squamocolumnar junction (75 genes) and the ectocervix squamous region (660 genes) [29], comes to reinforce this idea. Specifically, while genes characterizing ectocervical squamous population were enriched in all gynecological cancers (cervical 65 genes, endometrial 86 genes, vulvar 47 genes), squamocolumnar junction cells shared similarities with the upregulated genes in endometrial (7 genes) and cervical cancer patients (9 genes), but not with the vulvar cancer patients (3 genes), as shown in Fig 3A. This squamocolumnar junction group included genes such as complement factor B and H (*Cfb*, *Cfh*), which are involved in the regulation of immune reaction and interferon-induced protein 44 (*Ifi44l*), which is also activated in anti-viral response. Interestingly, *S100p* gene, which is associated with Cell Cycle, Cell Growth and Invasion and reported to be a novel independent predictor for poor prognosis in colorectal and hepatocellular carcinoma [30], was also found upregulated only in vulvar cancer samples in our study. However, despite the high levels of its expression found also in cervical cancer patients, it was not considered differentially expressed (*p* = 0.07), due to variation among the cancer samples. These data provide for the first time a direct comparison between gynecological cancers and this novel anatomical area, which may potentially host putative early initiating cancer events [29].

### Embryonic stem cells share common features with gynecological cancers

Considering the enrichment in stem cell signatures and based on recent findings on gene expression similarities and self-renewal properties between cancer and embryonic stem cells (ES), we detected significant enrichment of Myc and Polycomb (Prc) modules operating in embryonic stem cells [31], also in the cervical, endometrial and vulvar cancer patients (Fig 3B). From the three characterized modules in embryonic stem cells [31], Myc module was enriched in the upregulated population of all three gynecological cancer types studied here, while Prc was enriched in the downregulated differentially expressed genes of cervical and endometrial cancers. E-cadherin [32] and *Epcam* [33] are early markers and key genes in the reprogramming process involved in embryonic stem cell colony formation and establishment. Both genes were found to be upregulated in cervical and endometrial cancer (*Epcam* in cervical cancer, *p* = 0.14) but not in vulvar cancer patients. This finding is consistent with our observation so far, that endometrial and cervical cancers share a greater extent of common features (genes, gene signatures, altered biological processes) than with vulvar cancer. This may reflect the common embryonic origin of cervix and uterus.

### Identification of common multiple features in gynecological cancers

Regardless of the fact that the majority of the databases cover a wide variety of cancers and oncogenic signatures, in our study we selected representative studies from each cancer or pre-cancerous type that contained also normal tissue samples, for the identification of informative gynecological cancer-specific gene markers, and compared them with our data using the same methodology. Analysis of vulvar intraepithelial neoplasia (VIN) lesions [34,35], which represents a pre-cancerous stage of vulvar cancer, showed good concordance (~35%) with our previously characterized gene signature in vulvar cancer patients [6]. Vulvar intraepithelial neoplasia also showed upregulation of genes involved in Cell Cycle and Apoptosis, and downregulation of genes involved in Transcription and Development. Similar results were obtained from re-analyzing lymph nodes (LN) from squamous cell vulvar carcinoma [36] with ~28% concordance, when we compared the biological functions of the upregulated and downregulated genes. Cell Cycle, Apoptosis, Transcription and Developmental-related categories were enriched in the differentially expressed genes of LN (+) vs. LN (-), as in our study. The same categories were enriched in cervical cancer studies [4,5,37,38] and endometrial study [39], either in gene ontology analysis of biological functions or in pathway analysis. By extending our findings to other gynecological cancer studies, it was made obvious that there were common features among endometrial, vulvar and cervical cancer cells in terms of biological functions and pathway deregulation. Based on the small overlap between the genes differentially expressed in each study, we arrive to the conclusion that more than one network or cascade can lead to malignant transformation in vulvar, cervical and endometrial tissues.

### Identification of key transcription factors in gynecological cancer formation and novel modules in cervical cancer

**Fig 4:**
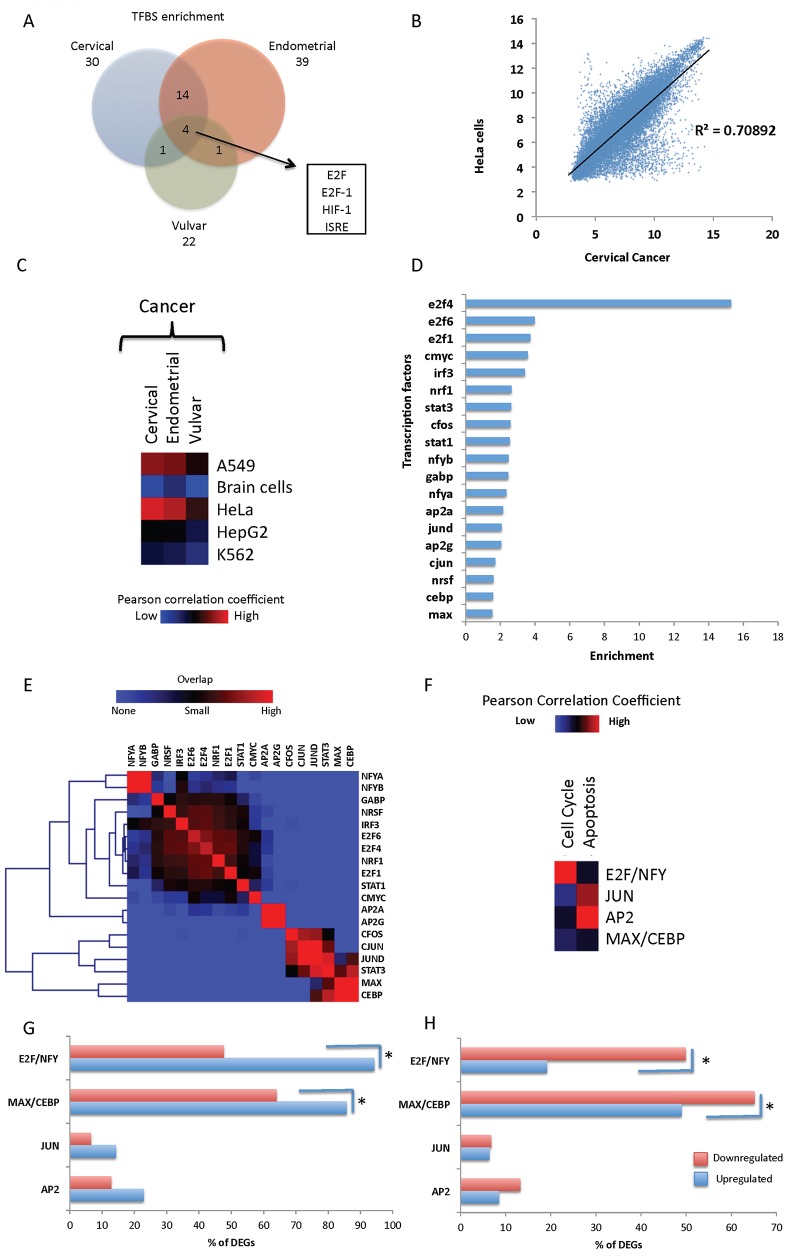
Identification, annotation of novel cervical cancer modules and comparison with embryonic stem cell modules using both in silico and in vivo studies (ENCODE) and correlation of module activity with apoptosis and cell cycle. A. Venn diagram of commonly transcription regulators found to be enriched near the TSS (-3000, +200 bp) of upregulated genes in each gynecological cancer. B. Scatter plot showing the correlation between the HeLa cell line and a cervical cancer array (r = 0.84). C. Heatmap of the correlation of the expression of all gynecological cancers and the corresponding normal samples, with the expression of HeLa, K562, A549, HepG2 and normal brain cells as calculated from more than three independent studies for each tissue or cell line. Accession numbers from all the studies are shown in S3 Table. D. Bar graph depicting the enrichment of annotated transcription regulators from ENCODE in HeLa cells in the upregulated genes in cervical cancer patients. E. Heatmap of transcription factor binding site overlap of transcription regulators enriched in cervical cancer upregulated genes (S4 Table). F. Heatmap showing the correlation of JUN and AP2 modules with apoptosis, and of E2F/NFY with cell cycle regulation. G. Bar graphs of the correlation of E2F/NFY and MAX/CEBP modules with Myc module for upregulated genes. H. Bar graphs of the correlation of E2F/NFY and MAX/CEBP modules with Prc module for downregulated genes.

Identification of transcription factor (TF) enrichment with PRIMA algorithm [40] in a narrow region around gene's transcription start site (TSS), extending from -1000 to +200 bp, led to the identification of 30, 39 and 22 transcription factors for cervical, endometrial and vulvar cancer, respectively. *E2f*, *E2f1*, *Hif-1* and *Isre* were the four transcription factors that were found enriched in all cancer types (Fig 4A), with *E2f1* (*p* = 0.038) and *Hif*-1 (*p* = 0.004) being also enriched in the 72 common upregulated genes (Fig 1A). Hypoxia-induced factor 1, *Hif*-1, upregulated in cervical and vulvar cancer, has been identified to respond to changes in oxygen levels in the cellular environment and mediating the effects of hypoxia. Hypoxia promotes the formation of blood vessels and contributes to the formation of cancer tumors in breast [10,41]. *E2f* transcription factor family is associated with cell cycle regulation targeting cyclin A2 (*Ccna2*, upregulated in cervical and endometrial carcinoma), cyclin D1 (*Ccnd1* upregulated in endometrial carcinoma), cyclin-dependent kinase 2 (*Cdk2*, upregulated in cervical cancer) and apoptosis-related genes such as *Casp3* (upregulated in cervical cancer) and *Casp8* (upregulated in endometrial cancer). In addition to these transcription regulators, other well-characterized factors such as *Nf-y*, *Stat1*, *Irfs* and *c-Myc*:*Max*, were also enriched in the 72 commonly upregulated genes. Interestingly, comparison with other studies on vulvar [34,35], cervical [4,5,37,38] and endometrial [39] cancer, disclosed *Hif-1* and *E2f* as key transcription regulators for the modulation of gene expression in cancer. Apart from these two transcription regulators, *Zf5* a known interactor of *Brca1* tumor suppressor [42], was also found enriched in all the previous studies used for comparison and in our vulvar cancer gene signature study [6]. Promoter analysis showed that there is a certain set of transcription factors that regulate the differential expression between normal and cancer samples in all gynecological cancers (Fig 4A).

Apart from the computational identification of transcription factor binding sites, access to the ChIP-seq experiments of ENCODE database [43], led us to investigate the correlation of the available transcription regulators that were found to be enriched in the upregulated genes in cervical cancer patients, using data from the HeLa cervical cancer cell line [44]. HeLa cells, which have been systematically studied with *next generation sequencing* (*NGS*) and microarray technologies [43,44], exhibited as expected, a high correlation with the cervical cancer expression profile (Fig 4B and S4 Table). In order to systematically investigate the extent of common features between HeLa cells and cervical cancer cell expression profile, we randomly selected three independent studies, which investigated the expression profile on the same microarray platform, and applied the same steps in analyzing the gene's expression levels. All arrays from the HeLa experiment, individually or averaged, exhibited high correlation mainly with cervical cancer cells (Fig 4C and S4 Table). Additional comparisons of the average expression profile of all three gynecological cancers with other well-studied cancer cell lines (A549, K562, HepG2) and normal tissues (brain), verified that HeLa cell line can faithfully simulate cervical cancer expression profile.

Furthermore, transcription factors *Stat1*, *Stat3*, *Cebp*, *E2f1*, *E2f4*, *E2f6*, *Fos*, *Myc*, *Ap2* and *Jun* were all enriched in the upregulated genes, with a relative ratio ranging from 1.5 to 15 (Fig 4D) for upregulated against downregulated genes (S2 Table), when annotated in a region of 5 kb around TSS (-2500 to +2500 bp) of the differentially expressed genes in cervical cancer patients. Analysis of the overlapping regions of these transcription regulators, revealed for the first time four novel distinct modules, which actually represent smaller sets of gene sub-signatures [31] (Fig 4E and S4 Table). The first module (E2f/NFY module) consists of *Nfy-A*, *Nfy-B*, *Gabp*, *Nrsf*, *Irf3*, and *E2f* transcription regulators, *Nrf1*, *Stat1* and *cMyc*, with most of them regulating transcription of cell cycle processes. Two additional novel modules consisting of *Ap2a* and *Ap2g* (AP2 module) and *Max* and *Cebp* (Max/Cebp module), respectively, segregated from the other factors, while a fourth cluster of transcription regulators with *cJun*, *Jund*, *cFos* and *Stat3* (JUN Module), was associated with inflammatory response. All modules were found to be significantly enriched in the upregulated genes, controlling expression of most cervical cancer gene signatures (701 out of 763 genes), while combination in pairs was more frequent by 2.5 to 10-fold in the upregulated population, leading to the conclusion that modules interact and cooperate for the activation of cervical cancer signature genes.

Despite the synergy and cooperation of the transcription regulators in each module, we then investigated the role of each factor and ranked them based on the percentage of genes they regulate in each of the biological functions affected in all gynecological cancers and embryonic stem cell modules. The E2F/NFY module was ranked as the first and main module for regulating cell cycle process, while the AP2 and JUN modules, were ranked as the main modules in apoptosis (Fig 4F). From the modules characterized in ES cells [31], Myc module showed correlation with both E2F/NFY and MAX/CEBP (*p* < 0.05, *χ* ^2^-test), suggesting a possible synergy among the individual transcription regulators forming these modules on the regulation of expression of the cervical cancer signature genes. In the opposite direction, the Prc module, which is the main repressing complex in embryonic stem cells [31], is more frequently observed in the absence of the E2F/NFY and MAX/CEBP modules (*p* < 0.05, *χ* ^2^-test). With this analysis, we observed a positive correlation between both E2F/NFY and MAX/CEBP modules with the Myc module (Fig 4G), and a negative correlation with the Prc module (Fig 4H).

**Fig 5:**
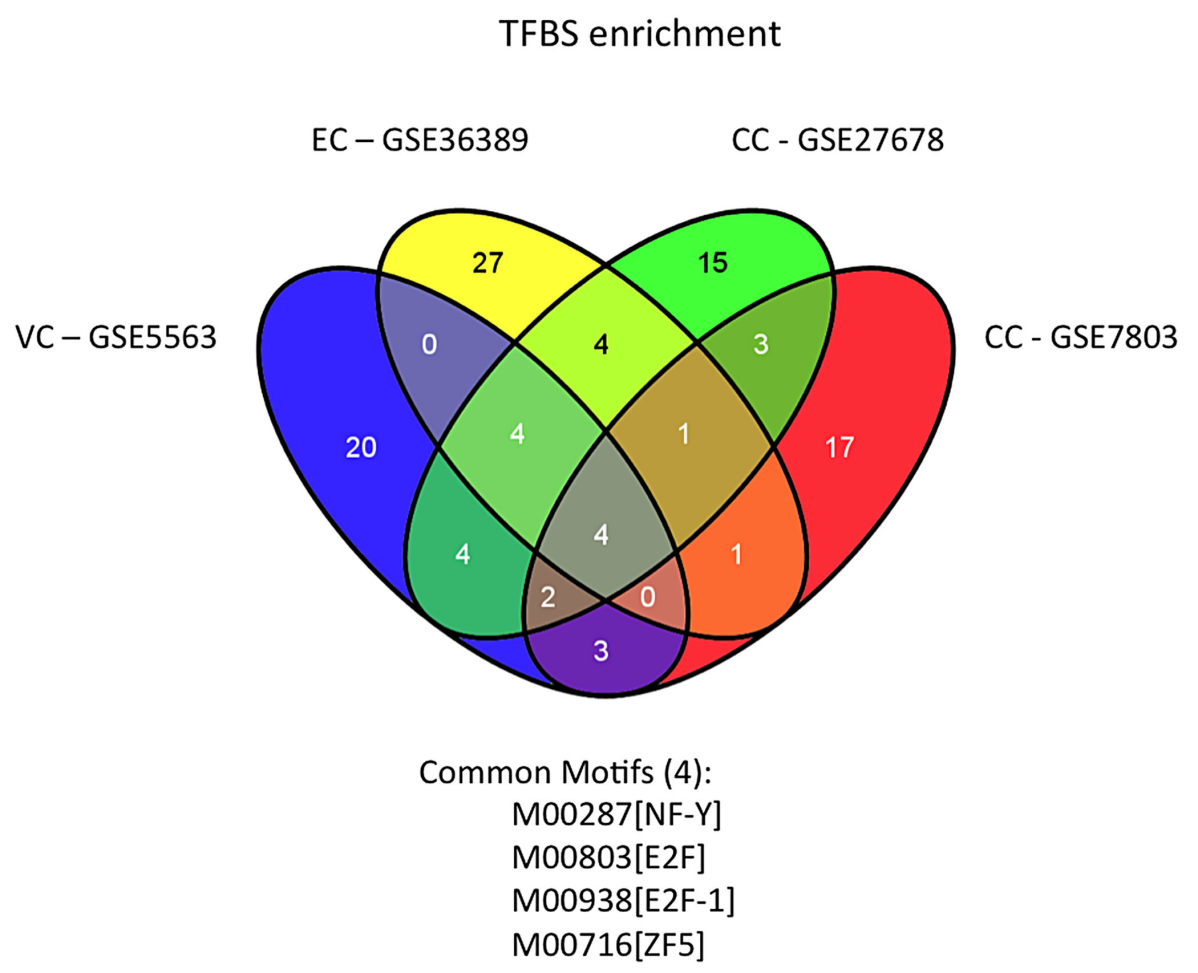
Transcription factor binding analysis in various gynecological cancer expression profiles revealed enrichment of E2F/NFY module. Analysis of microarray expression profile studies conducted in various gynecological cancers, revealed that E2F/NFY module is enriched in all gynecological cancers. Genes that were differentially expressed in GSE5563, GSE7803, GSE27678 and GSE36389 datasets, utilizing the same methodology as described in our study, were used for TFBS enrichment analysis. Common transcription regulators identified in all gynecological cancers exhibited a significant enrichment of E2F/NFY module in gynecological cancers.

Both informatics analysis of the motifs of known transcription regulators and experimental evidence from ENCODE, identified key components of the transcription regulation factories of gynecological cancer development. Identification of *E2f*, *Nfy* and other transcription factors, led us to the construction of novel transcriptional modules, which regulate the activation of cancer-related genes. E2F/NFY module was the only module found enriched in all gynecological cancers in our study (Fig 5). Though few expression profile studies have been performed in gynecological cancers, we were able to identify the same transcription factors enriched in the upregulated genes (Fig 5), in four other studies from (GSE5563 [34], GSE7803 [5], GSE27678 [38], GSE36389 [39]), using the same approach.

## Discussion

The systematic comparison among the three gynecological cancers, resulted in the identification of 193 genes being commonly regulated in all three types of cancers. Despite their small overlap regarding differentially expressed genes, biological function classification revealed common features among the three gynecological cancers. This deregulation concerned upregulation of cell cycle process [15,28,34], activation of apoptosis-related genes and downregulation of genes involved in transcriptional activity.

Furthermore, comparisons highlighted the extensive similarity between cervical and endometrial cancer regarding the differentially expressed genes, the biological processes affected, and the transcription factor binding sites. This similarity was less pronounced in vulvar carcinoma. This pattern was recapitulated when we compared gene expression profiles from the recent genomic classification on endometrial carcinoma by The Cancer Genome Atlas Research (TCGA) Network with our gynecological cancers [9]. Endometrial and cervical cancer profiles correlated mainly with the expression profile of *POLE* and MSI subcategories [9], while vulvar carcinoma was found to deviate from those profiles. Even though the differences and the variable expression of driver genes for cancer formation (*ErbB2*, *Akt3*, *Pik3ca*) was documented in cervical, endometrial and vulvar carcinoma, the positive correlation of the differentially expressed genes of the three types of gynecological cancers with modules (Myc), pathways (Wnt, ErbB, Toll-like receptors) and biological processes lead us to assume that there is more than one operating gene network that regulates the same functions and pathways. Another crucial component controlling gene regulation is microRNA. Though very few studies [45,46] have extensively studied microRNA profiling in ovarian and cervical cancer, no additional experimental information was found for the three gynecological cancers to complement our findings at the moment.

Furthermore, downregulation of *ErbB*-2 and *ErbB*-3 genes is associated with neuro-degenerative diseases such as multiple sclerosis and Alzheimer's disease [47]. This finding highlights the presence of common routes leading to diverse disorders sharing common primitive master pathways. Thus, activation or repression of a different set of genes in one process, can lead to similar downstream effects. This is verified from the small gene overlap from the numerous different gene signatures annotated to the same disease [18,19]. In order to reduce the variance due to different statistical approaches and methodologies utilized for microarray analysis, we performed the same methodology, as in our study, in a number of selected gynecological cancer studies and verified the small overlap (20–38%) between gene signatures. From these comparisons, we noted the common biological functions in most of the studies and identified a set of transcription regulators (*E2f*, *Hif-1*) as key regulators in endometrial, vulvar and cervical cancer patients. Though no meta-analysis was performed, random selection of various expression profile studies in gynecological cancer increased the validity of our findings regarding the processes, pathways and transcription regulators.

It should be noted that either upregulation or downregulation of a key gene such as *Akt3* can cause deregulation of one or more pathways and lead to malignancy [48]. In our study, *Akt* signaling pathway from BIOCARTA and PI3K-Akt pathway from PANTHER database, were found enriched only in endometrial cancer as it has been previously noted [9]. Additionally, DNA damage control and DNA repair mechanisms are mainly downregulated during early tumor development. *Arid1a* loss has been shown to occur in high-grade endometrial cancers [28], leading to a series of mutations, which occur following *Arid1a* downregulation. The downregulation of *Arid1a* in endometrial and vulvar cancer in the series of patients of our study, is consistent with these findings.

Also in our study, we tested for the first time the expression profile of gynecological cancers to that of a recently identified quite discrete cuboidal cell population of squamocolumnar junction (SJ) cells with embryonic-like features. These cells are considered to be implicated in the pathogenesis of cervical cancer and represent a specific target of HPV [29]. Thus, this cell population has been proposed as the cell of origin of cervical cancer and its precursors [49].

Interestingly, in our study we identified in the squamocolumnar junction cells several active markers of carcinogenesis, which were also enriched in cervical and endometrial but not in vulvar carcinoma. These data support the notion that this population actually represents a premalignant state, which can evolve to cancer upon infection by HPV. This progression seems to be partially attributed to the permissiveness of the cells to viral entry, due to the documented altered expression of α-defensin 5 by these cells, compared to the ectocervical, vaginal, and vulvar neoplasia [50].

It is also of interest that squamocolumnar junction cells can define distinct clinically relevant subsets of low-grade squamous intraepithelial lesions [51]. Recent immunohistochemical studies of specific markers of the behavior of these cells during neoplasia, suggest that HPV infection of the cuboidal squamocolumnar junction cells initiates the outgrowth of basally oriented neoplastic progeny with a progressive loss of the embryonic markers [49]. This pattern was observed in high-grade squamous intraepithelial lesions (HSIL) but not in low grade ones (LSIL), a fact that suggests that in the latter cases, the infection occurs in metaplastic progeny rather than in the original squamocolumnar junction cells. Corroborating this notion, recent clinical studies [52] documented the beneficial effect of squamocolumnar junction excision in reducing the risk of developing new cervical intraepithelial neoplasia 2/3 lesions. Further comparative studies on the transcriptomics of this cell population and of early cervical carcinogenesis [53], are anticipated to offer significant insights into the early events of cervical carcinogenesis.

Finally, with the use of ChIP-seq experiments from ENCODE [43] in the HeLa cervical cancer cell line, we identified for the first time four novel modules operating in our cervical cancer samples. Correlation analysis revealed synergy between those groups of transcription regulators, while certain modules were annotated to specific biological processes. The E2F/NFY module was mainly enriched in cell cycle processes while the JUN and MAX/CEBP modules were enriched in apoptotic processes. We also noted that beside the synergy of these modules, E2F/NFY and MAX/CEBP correlated with Myc module, while Prc module was present in genes where factors from E2F/NFY and MAX/CEBP modules were absent. These data strongly imply that there is a common transcriptional network based on *E2f*, *Nfy* and other transcription regulators, in a form of cooperating modules regulating the main cellular features, such as cell cycle, apoptosis, transcription and development. Aberrant operation of those modules, is required for the gradual transformation into a cancer cell via intraepithelial neoplasia of a normal squamocolumnar junction cell or epithelial cell in the case of cervical and vulvar cancers, respectively, or of an epithelial cell in the case of endometrial cancer.

In summary, our novel data may have implications in the field of human carcinogenesis, since the validity of these novel modules can be tested in other types of cancers of different origin, for the formulation of comprehensive models of carcinogenesis, which may lead eventually to rational preventive and therapeutic strategies for precise targeting of common and unique altered regulatory mechanisms operating in several types of cancers [3].

## Methods

### Tissue and sample selection

A total of 35 snap-frozen samples were analyzed, derived from 18 cancer patients (5 patients with cervical cancer, 7 patients with endometrial cancer and 6 patients with vulvar cancer) and from 17 normal control samples (5 normal cervical samples, 5 normal endometrial samples and 7 normal vulvar samples) from patients undergoing surgery for benign gynecological diseases (fibroids, ovarian benign cyst or uterine prolapse). The tumor samples were classified according to the new 2009 FIGO staging system [54] and the histological classification system of WHO. All tissues were obtained using verbal informed consent, recorded in the participants list, following the approval (No. 6/16-11-2005) of this study by the Institutional Ethical Committee of the Alexandra Hospital. The patients were recruited at the Alexandra Hospital in Athens, from 2006 and onwards. None of the patients had received any preoperative chemotherapy or irradiation treatment.

### Microarray analysis

Total RNA was extracted from normal controls and cancer patients from cervix, endometrium and vulvar tissues and was hybridized on Affymetrix HG133 A 2.0 microarray chips corresponding to more than 14,500 uniquely represented genes (NetAffx 32). A total of 35 samples were used to identify potential biomarkers and signatures in each type of cancer. The data were analyzed with the R language (version 3.0.2) and bioconductor package (version 2.13) [55]. The RMA algorithm [56] and log2 transformation were used for background correction and normalization of the data. For those genes that were represented in more than one probe, only probes with the highest average value across all arrays were kept. In order to identify differentially expressed genes, we performed a Student's t-test in unlogged data between normal and cancer tissues, and those genes with a value of *p* < 0.05 and a fold change (±) greater than 1.5, were considered significant. The microarray data (GSE63678) were submitted to GEO [57].

### Functional annotation and pathway analysis

Gene ontology classification and pathway annotation was performed with Expander 5.2 [21,22] and DAVID knowledgebase 6.7 [23,24] combining information from KEGG, REACTOME, BIOCARTA and PANTHER pathway databases. Additional comparison of the biological processes was performed with the use of Comparative GO [17] using all the upregulated and downregulated genes with their corresponding fold changes in each gynecological cancer type. Transcription factor binding site (TFBS) enrichment analysis was performed with both PRIMA algorithm and DAVID knowledgebase 6.7 [23,24] and transcription regulators were annotated when a motif was found close to a gene's transcription start site (TSS) in a region of 1200 bp (-1000, +200 bp). Hypergeometric test was performed in order to identify significant enrichment while Fisher’s exact test was performed in DAVID knowledgebase 6.7 [23,24].

### Gene signature analysis

GeneSigDB 4.0 [19] and oncogenic signatures from GSEA 2.0.14 [18] were used for comparing our findings with other known signatures. We selected as overlapping signatures, gene lists with more than 5 genes in common and *p* < 0.01. In each database, all studies were merged based on the tissue of origin of the cancer, regardless of the effect or of the treatment in each study. Selected microarray studies from each gynecological cancer type consisted of both cancer and normal tissue samples. RMA normalization and Student’s t-test was performed and genes with a value of p < 0.05 and a fold change (±) greater than 1.5, were considered to be differentially expressed. GSE27678, GSE9750, GSE7803, GSE28442, GSE5563, and GSE36389, were the accession codes selected for gene ontology, pathway and signature comparison.

### Modules

In order to identify modules in cervical carcinoma, we examined the overlaps between the '.bed' files for each transcription regulator given in ENCODE [43] with the use of BEDTools 2.17.0 [58]. Based on the number of overlapping regions for each transcription regulator with all the other transcription regulators, we performed normalization based on the number of binding sites for each transcription regulator and then performed unsupervised hierarchical clustering in order to identify the cervical cancer modules. Finally, a gene was considered regulated by a module, when at least 2 transcription regulators were bound around its TSS (-2500, +2500 bp).

### Statistical analysis

Chi-square test with or without Yates correction was performed when needed. Hypergeometric test was also performed for enrichment identification between embryonic stem cell modules and the newly identified regions of cervix with our differentially expressed genes. Correlation was measured with Pearson correlation coefficient. Unsupervised hierarchical cluster analysis was performed with tMEV 4.8.1 software with Pearson correlation coefficient and average linkage method.

## Supporting Information

S1 Fig Principal component analysis of microarray experiments in the three gynecological cancers and their normal controls. Principal component analysis in two axis, depicts the separation of normal and cancer samples based on the differentially expressed genes in cervical, endometrial and vulvar samples.
(TIF)

S2 Fig Activation of virus response genes in cervical cancer and downregulation of ARID1A in endometrial and vulvar cancer. A. Heatmap of normalized cervical cancer samples showing differentially expressed genes involved in ‘response to virus’. B. Average ARID1A expression levels in the three gynecological cancers and their corresponding normal samples. Though ARID1A was found downregulated (*p* <0.05) in endometrial and vulvar cancer, vulvar cancer samples exhibited greater reduction (fold change = -1.7 in vulvar cancer vs -1.3 in endometrial cancer). For significant differences with *p* < 0.05, an asterisk (*) was used for annotation.
(TIF)

S3 Fig Comparison of network terms common in all gynecological cancers. A. Venn diagram comparing the terms in network formation from IPA software in upregulated genes. B. Venn diagrams of downregulated genes in the three gynecological cancers of the study. Below are shown the common network terms in each comparison. The categories that are unique in upregulated and downregulated common network terms are shown in bold.
(TIF)

S4 Fig Top networks in common differentially expressed genes in all gynecological cancer expression profiles. Networks formed with IPA using the common regulated genes from all gynecological cancers (193 genes). A. Cell cycle-related network. B. Cancer and Cell death and Survival-related networks were among the top three networks that exhibited the highest score.
(TIF)

S1 Table Patient clinopathological features. Clinicopathological features of the patients and normal controls of the study. Cancer cases were staged according to the 2009 FIGO staging guidelines [52].
(DOC)

S2 Table List of differentially expressed genes in all gynecological cancers with their gene ontology (GO) and pathway classification. List of differentially expressed genes with fold change, average expression value and categorization in upregulated and downregulated expression. Gene ontology (GO) analysis for the differentially expressed genes (upregulated and downregulated) of each cancer versus genome, pathway analysis, TFBS analysis for both upregulated and downregulated genes. gene signature analysis information and lists, are shown in separate spreadsheets.
(XLS)

S3 Table Comparison of enrichment between Biological Processes in Cervical, Endometrial and Vulvar Cancer. We present biological proceses common in all gynecological cancers in the upregulated and downregulated genes that were found to be enriched in one gynecological cancer at least 2 times more that the other gynecological cancers. In the upregulated genes we focused in cell cycle, transcriptional and apoptosis related processes while in the downregulated gene population we focused in developmental related processes.
(XLSX)

S4 Table Genes and expression values from various studies used for comparison with our gynecological cancers. In the first spreadsheet (ST4__FIGURE4B) we present the normalized expression values from Cervical cancer and HeLa cells from randomly selected microarrays used for calculation of the correlation between HeLa and Cervical cancer cells in Fig 4B. ST4__FIGURE4C spreadsheet contains the average expression values from the microarray studies used for Fig 4C. ST4_FIGURE4E spreadsheet contains all the differentially expressed genes from our gynecological studies which are bound by one of the transcription factors studied in ENCODE in HeLa cell line. The values 0 and 1 represent the absence (0) or the existence (1) of one transcription factor near the promoter of the selected gene. GEO LINKS spreadsheet contains all the GEO accessions, tissue types and links used for the transcription factor binding analysis presented in Fig 5.
(XLSX)

S5 Table Gene Expression Omnibus (GEO) submitted gynecological studies. List of GEO accession codes used for comparative analysis of the expression profile of cervical cancer samples with HeLa, A549, K562, HepG2 and normal brain cells.
(DOC)

S6 Table List of modules and their genes in cervical cancer. Modules identified in cervical cancer samples. Each spreadsheet contains the differentially expressed genes regulated by the identified set of transcription factors found to co-occupy their promoters.
(XLS)

## References

[1] U.S. Cancer Statistics Working Group. United States Cancer Statistics: 1999–2011 Incidence and Mortality Web-based Report. U.S. Department of Health and Human Services, Centers for Disease Control and Prevention and National Cancer Institute, Atlanta, Ga (2014). Available: www.cdc.gov/uscs. Accessed 3 March 2015.

[2] Slack JMW. Essential Developmental Biology. 2nd ed. Oxford: Blackwell Publishing; 2006.

[3] Yang Y, Han L, Yuan Y, Li J, Hei N, Liang H. Gene co-expression network analysis reveals common system-level properties of prognostic genes across cancer types. Nat Commun. 2014;5:3231 10.1038/ncomms4231 24488081 PMC3951205

[4] Winder DM, Chattopadhyay A, Muralidhar B, Bauer J, English WR, Zhang X, et al Overexpression of the oncostatin M receptor in cervical squamous cell carcinoma cells is associated with a pro-angiogenic phenotype and increased cell motility and invasiveness. J Pathol. 2011;225:448–462. 10.1002/path.2968 21952923

[5] Zhai Y, Kuick R, Nan B, Ota I, Weiss SJ, Trimble CL, et al Gene expression analysis of preinvasive and invasive cervical squamous cell carcinomas identifies HOXC10 as a key mediator of invasion. Cancer Res. 2007;67:10163–10172. 17974957 10.1158/0008-5472.CAN-07-2056

[6] Pappa KI, Jacob-Hirsch J, Vlachos GD, Christodoulou I, Partsinevelos G, Amariglio N, et al Expression profiling of vulvar carcinoma: clues for deranged extracellular matrix remodeling and effects on multiple signaling pathways combined with discrete patient subsets. Transl Oncol. 2011;4:301–313. 21966547 10.1593/tlo.11148 PMC3162305

[7] Choschzick M, Hess S, Tennstedt P, Holst F, Bohlken H, Gieseking F, et al Role of cyclin D1 amplification and expression in vulvar carcinomas. Hum Pathol. 2012;43:1386–1393. 10.1016/j.humpath.2011.11.014 22406359

[8] Ojesina AI, Lichtenstein L, Freeman SS, Pedamallu CS, Imaz-Rosshandler I, Pugh TJ, et al Landscape of genomic alterations in cervical carcinomas. Nature. 2014;506:371–375. 10.1038/nature12881 24390348 PMC4161954

[9] Cancer Genome Atlas Research Network. Integrated genomic characterization of endometrial carcinoma. Nature. 2013;497:67–73. 10.1038/nature12113 23636398 PMC3704730

[10] Cancer Genome Atlas Research Network. Comprehensive molecular portraits of human breast tumours. Nature. 2012;490:61–70. 10.1038/nature11412 23000897 PMC3465532

[11] Cancer Genome Atlas Research Network. Integrated genomic analyses of ovarian carcinoma. Nature. 2011;474:609–615. 10.1038/nature10166 21720365 PMC3163504

[12] Alisoltani A, Fallahi H, Ebrahimi M, Ebrahimi M, Ebrahimie E. Prediction of potential cancer-risk regions based on transcriptome data: towards a comprehensive view. PLoS One. 2014;9:e96320 10.1371/journal.pone.0096320 24796549 PMC4010480

[13] Peng L, Bian XW, Li DK, Xu C, Wang GM, Xia QY, et al Large-scale RNA-seq transcriptome analysis of 4043 cancers and 548 normal tissue controls across 12 TCGA cancer types. Sci Rep. 2015;5:13413 10.1038/srep13413 26292924 PMC4544034

[14] Martinez E, Yoshihara K, Kim H, Mills GM, Trevi–o V, Verhaak RG. Comparison of gene expression patterns across 12 tumor types identifies a cancer supercluster characterized by TP53 mutations and cell cycle defects. Oncogene. 2015;34:2732–2740. 10.1038/onc.2014.216 25088195 PMC4317393

[15] Mine KL, Shulzhenko N, Yambartsev A, Rochman M, Sanson GF, Lando M, et al Gene network reconstruction reveals cell cycle and antiviral genes as major drivers of cervical cancer. Nat Commun. 2013;4:1806 10.1038/ncomms2693 23651994 PMC4237593

[16] Hoadley KA, Yau C, Wolf DM, Cherniack AD, Tamborero D, Ng S, et al Multiplatform analysis of 12 cancer types reveals molecular classification within and across tissues of origin. Cell. 2014;158:929–944. 10.1016/j.cell.2014.06.049 25109877 PMC4152462

[17] Fruzangohar M, Ebrahimie E, Ogunniyi AD, Mahdi LK, Paton JC, Adelson DL. Correction: Comparative GO: a web application for comparative gene ontology and gene ontology-based gene selection in bacteria. PLoS One. 2015;10:e0125537 10.1371/journal.pone.0125537 25884626 PMC4401681

[18] Subramanian A, Tamayo P, Mootha VK, Mukherjee S, Ebert BL, Gillette MA, et al Gene set enrichment analysis: a knowledge-based approach for interpreting genome-wide expression profiles. Proc Natl Acad Sci U S A. 2005;102:15545–15550. 16199517 10.1073/pnas.0506580102 PMC1239896

[19] Culhane AC, Schröder MS, Sultana R, Picard SC, Martinelli EN, Kelly C, et al GeneSigDB: a manually curated database and resource for analysis of gene expression signatures. Nucleic Acids Res. 2012;40:D1060–1066. 10.1093/nar/gkr901 22110038 PMC3245038

[20] Walboomers JM, Jacobs MV, Manos MM, Bosch FX, Kummer JA, Shah KV, et al Human papillomavirus is a necessary cause of invasive cervical cancer worldwide. J Pathol. 1999;189:12–19. 10451482 10.1002/(SICI)1096-9896(199909)189:1<12::AID-PATH431>3.0.CO;2-F

[21] Ulitsky I, Maron-Katz A, Shavit S, Sagir D, Linhart C, Elkon R, et al Expander: from expression microarrays to networks and functions. Nat Protoc. 2010;5:303–322. 10.1038/nprot.2009.230 20134430

[22] Shamir R, Maron-Katz A, Tanay A, Linhart C, Steinfeld I, Sharan R, et al EXPANDER—an integrative program suite for microarray data analysis. BMC Bioinformatics. 2005;6:232 16176576 10.1186/1471-2105-6-232 PMC1261157

[23] Huang da W, Sherman BT, Lempicki RA. Systematic and integrative analysis of large gene lists using DAVID bioinformatics resources. Nat Protoc. 2009;4:44–57. 10.1038/nprot.2008.211 19131956

[24] Huang da W, Sherman BT, Lempicki RA. Bioinformatics enrichment tools: paths toward the comprehensive functional analysis of large gene lists. Nucleic Acids Res. 2009;37:1–13. 10.1093/nar/gkn923 19033363 PMC2615629

[25] Clevers H, Nusse R. Wnt/β-catenin signaling and disease. Cell. 2012;149:1192–1205. 10.1016/j.cell.2012.05.012 22682243

[26] Lustig B, Jerchow B, Sachs M, Weiler S, Pietsch T, Karsten U, et al Negative feedback loop of Wnt signaling through upregulation of conductin/axin2 in colorectal and liver tumors. Mol Cell Biol. 2002;22:1184–1193. 11809809 10.1128/MCB.22.4.1184-1193.2002 PMC134640

[27] Nusse R, Varmus HE. Wnt genes. Cell. 1992;69:1073–1087. 1617723 10.1016/0092-8674(92)90630-u

[28] Bosse T, ter Haar NT, Seeber LM, v Diest PJ, Hes FJ, Vasen HF, et al Loss of ARID1A expression and its relationship with PI3K-Akt pathway alterations, TP53 and microsatellite instability in endometrial cancer. Mod Pathol. 2013;26:1525–1535. 10.1038/modpathol.2013.96 23702729

[29] Herfs M, Yamamoto Y, Laury A, Wang X, Nucci MR, McLaughlin-Drubin ME, et al A discrete population of squamocolumnar junction cells implicated in the pathogenesis of cervical cancer. Proc Natl Acad Sci U S A. 2012;109:10516–10521. 10.1073/pnas.1202684109 22689991 PMC3387104

[30] Yuan RH, Chang KT, Chen YL, Hsu HC, Lee PH, Lai PL, et al S100P expression is a novel prognostic factor in hepatocellular carcinoma and predicts survival in patients with high tumor stage or early recurrent tumors. PLoS One. 2013;8(6):e65501 10.1371/journal.pone.0065501 23785431 PMC3681902

[31] Kim J, Woo AJ, Chu J, Snow JW, Fujiwara Y, Kim CG, et al A Myc network accounts for similarities between embryonic stem and cancer cell transcription programs. Cell. 2010;143:313–324. 10.1016/j.cell.2010.09.010 20946988 PMC3018841

[32] Soncin F, Ward CM. The function of e-cadherin in stem cell pluripotency and self-renewal. Genes (Basel). 2011;2:229–259. 24710147 10.3390/genes2010229 PMC3924836

[33] Ng VY, Ang SN, Chan JX, Choo AB. Characterization of epithelial cell adhesion molecule as a surface marker on undifferentiated human embryonic stem cells. Stem Cells. 2010;28:29–35. 10.1002/stem.221 19785009

[34] Santegoets LA, van Baars R, Terlou A, Heijmans-Antonissen C, Swagemakers SM, van der Spek PJ, et al Different DNA damage and cell cycle checkpoint control in low- and high-risk human papillomavirus infections of the vulva. Int J Cancer. 2012;130:2874–2885. 10.1002/ijc.26345 21815142

[35] Santegoets LA, Seters Mv, Helmerhorst TJ, Heijmans-Antonissen C, Hanifi-Moghaddam P, Ewing PC, et al HPV related VIN: highly proliferative and diminished responsiveness to extracellular signals. Int J Cancer. 2007;121:759–766. 17471573 10.1002/ijc.22769

[36] Kowalewska M, Radziszewski J, Goryca K, Bujko M, Oczko-Wojciechowska M, Jarzab M, et al Estimation of groin recurrence risk in patients with squamous cell vulvar carcinoma by the assessment of marker gene expression in the lymph nodes. BMC Cancer. 2012;12:223 10.1186/1471-2407-12-223 22673103 PMC3414830

[37] Scotto L, Narayan G, Nandula SV, Arias-Pulido H, Subramaniyam S, Schneider A, et al Identification of copy number gain and overexpressed genes on chromosome arm 20q by an integrative genomic approach in cervical cancer: potential role in progression. Genes Chromosomes Cancer. 2008;47:755–765. 10.1002/gcc.20577 18506748

[38] Ng G, Winder D, Muralidhar B, Gooding E, Roberts I, Pett M, et al Gain and overexpression of the oncostatin M receptor occur frequently in cervical squamous cell carcinoma and are associated with adverse clinical outcome. J Pathol. 2007;212:325–334. 17516585 10.1002/path.2184

[39] Orchel J, Witek L, Kimsa M, Strzalka-Mrozik B, Kimsa M, Olejek A, et al Expression patterns of kinin-dependent genes in endometrial cancer. Int J Gynecol Cancer. 2012;22:937–944. 10.1097/IGC.0b013e318259d8da 22706224

[40] Elkon R, Linhart C, Sharan R, Shamir R, Shiloh Y. Genome-wide in silico identification of transcriptional regulators controlling the cell cycle in human cells. Genome Res. 2003;13:773–780. 12727897 10.1101/gr.947203 PMC430898

[41] Ravi R, Mookerjee B, Bhujwalla ZM, Sutter CH, Artemov D, Zeng Q, et al Regulation of tumor angiogenesis by p53-induced degradation of hypoxia-inducible factor 1alpha. Genes Dev. 2000;14:34–44. 10640274 PMC316350

[42] Gorski JJ, Savage KI, Mulligan JM, McDade SS, Blayney JK, Ge Z, et al Profiling of the BRCA1 transcriptome through microarray and ChIP-chip analysis. Nucleic Acids Res. 2011;39:9536–9548. 10.1093/nar/gkr679 21880590 PMC3239190

[43] ENCODE Project Consortium. The ENCODE (ENCyclopedia Of DNA Elements) Project. Science. 2004;306:636–640. 15499007 10.1126/science.1105136

[44] Landry JJ, Pyl PT, Rausch T, Zichner T, Tekkedil MM, Stütz AM, et al The genomic and transcriptomic landscape of a HeLa cell line. G3 (Bethesda). 2013;3:1213–1224. 23550136 10.1534/g3.113.005777 PMC3737162

[45] Pereira PM, Marques JP, Soares AR, Carreto L, Santos MA. MicroRNA expression variability in human cervical tissues. PLoS One. 2010;5(7):e11780 10.1371/journal.pone.0011780 20668671 PMC2909898

[46] Iorio MV, Visone R, Di Leva G, Donati V, Petrocca F, Casalini P, et al MicroRNA signatures in human ovarian cancer. Cancer Res. 2007;67(18):8699–8707. 17875710 10.1158/0008-5472.CAN-07-1936

[47] Bublil EM, Yarden Y. The EGF receptor family: spearheading a merger of signaling and therapeutics. Curr Opin Cell Biol. 2007 4;19: 124–134. 17314037 10.1016/j.ceb.2007.02.008

[48] Altomare DA, Testa JR. Perturbations of the AKT signaling pathway in human cancer. Oncogene. 2005;24:7455–7464. 16288292 10.1038/sj.onc.1209085

[49] Herfs M, Vargas SO, Yamamoto Y, Howitt BE, Nucci MR, Hornick JL, et al A novel blueprint for 'top down' differentiation defines the cervical squamocolumnar junction during development, reproductive life, and neoplasia. J Pathol. 2013;229:460–468. 10.1002/path.4110 23007879

[50] Hubert P, Herman L, Roncarati P, Maillard C, Renoux V, Demoulin S, et al Altered α-defensin 5 expression in cervical squamocolumnar junction: implication in the formation of a viral/tumour-permissive microenvironment. J Pathol. 2014;234:464–477. 10.1002/path.4435 25196670

[51] Herfs M, Parra-Herran C, Howitt BE, Laury AR, Nucci MR, Feldman S, et al Cervical squamocolumnar junction-specific markers define distinct, clinically relevant subsets of low-grade squamous intraepithelial lesions. Am J Surg Pathol. 2013;37:1311–1318. 10.1097/PAS.0b013e3182989ee2 24076771 PMC3905241

[52] Herfs M, Somja J, Howitt BE, Suarez-Carmona M, Kustermans G, Hubert P, et al Unique recurrence patterns of cervical intraepithelial neoplasia after excision of the squamocolumnar junction. Int J Cancer. 2015;136:1043–1052. 10.1002/ijc.28978 24839092

[53] Thomas A, Mahantshetty U, Kannan S, Deodhar K, Shrivastava SK, Kumar-Sinha C, et al Expression profiling of cervical cancers in Indian women at different stages to identify gene signatures during progression of the disease. Cancer Med. 2013;2:836–848. 10.1002/cam4.152 24403257 PMC3892388

[54] Abu-Rustum NR, Zhou Q, Iasonos A, Alektiar KM, Leitao MM Jr, Chi DS, et al The revised 2009 FIGO staging system for endometrial cancer: should the 1988 FIGO stages IA and IB be altered? Int J Gynecol Cancer. 2011;21:511–516. 10.1097/IGC.0b013e31820cc305 21436699 PMC3870338

[55] Gentleman RC, Carey VJ, Bates DM, Bolstad B, Dettling M, Dudoit S, et al Bioconductor: open software development for computational biology and bioinformatics. Genome Biol. 2004;5:R80 15461798 10.1186/gb-2004-5-10-r80 PMC545600

[56] Bolstad BM, Irizarry RA, Astrand M, Speed TP. A comparison of normalization methods for high density oligonucleotide array data based on variance and bias. Bioinformatics. 2003;19:185–193. 12538238 10.1093/bioinformatics/19.2.185

[57] Barrett T, Wilhite SE, Ledoux P, Evangelista C, Kim IF, Tomashevsky M, et al NCBI GEO: archive for functional genomics data sets—update. Nucleic Acids Res. 2013;41:991–995. 10.1093/nar/gks1193 PMC3531084 23193258

[58] Quinlan AR, Hall IM. BEDTools: a flexible suite of utilities for comparing genomic features. Bioinformatics. 2010;26:841–842. 10.1093/bioinformatics/btq033 20110278 PMC2832824

