# Supplementary figures and images for: Profiling of Discrete Gynecological Cancers Reveals Novel Transcriptional Modules and Common Features Shared by Other Cancer Types and Embryonic Stem Cells
Source: PLoS One. 2015 Nov 11;10(11):e0142229. doi: 10.1371/journal.pone.0142229 (PMC4641642; doi:10.1371/journal.pone.0142229)

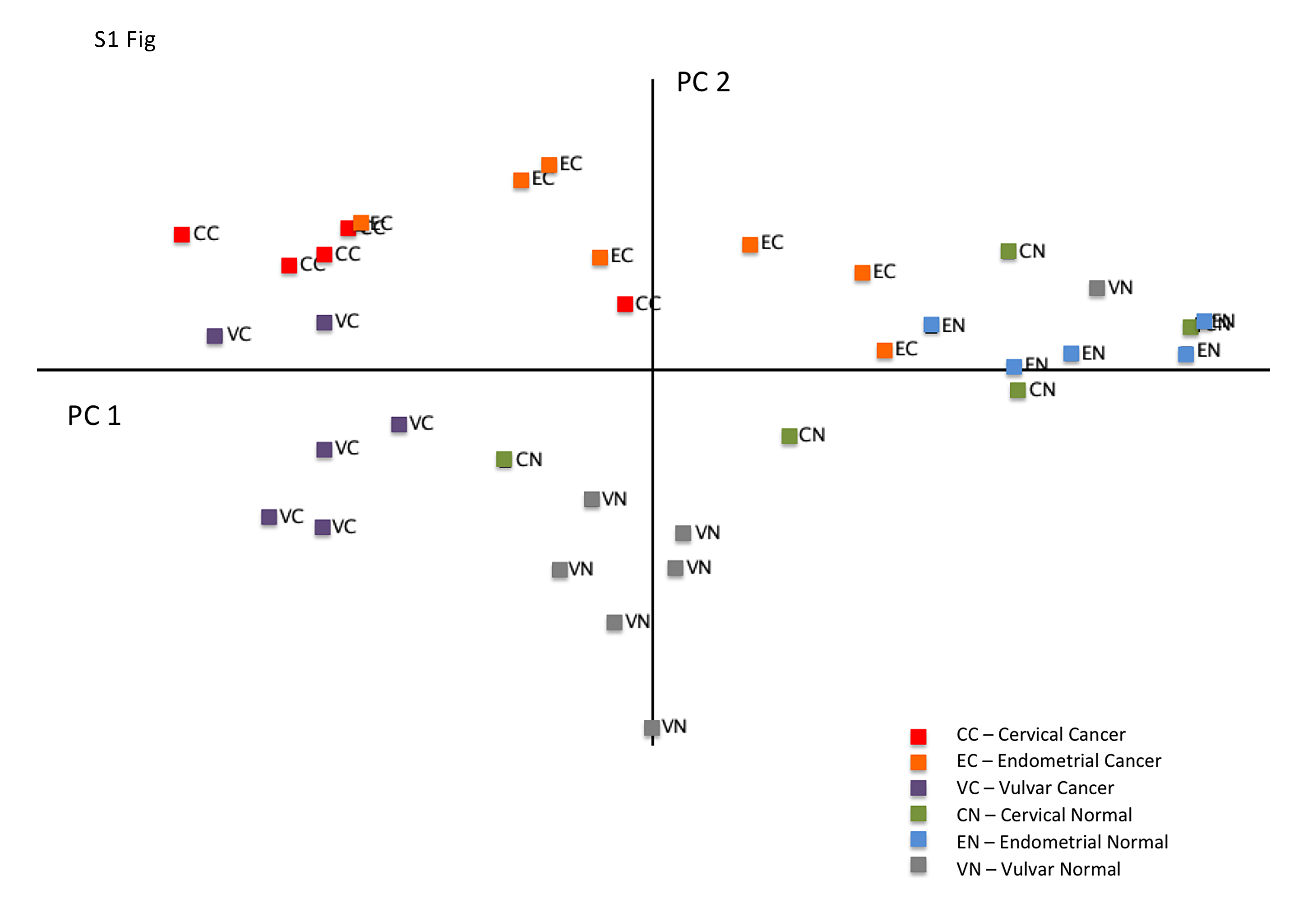

Supplement: S1 Fig — Principal component analysis in two axis, depicts the separation of normal and cancer samples based on the differentially expressed genes in cervical, endometrial and vulvar samples. (TIF) [file pone.0142229.s001.tif]

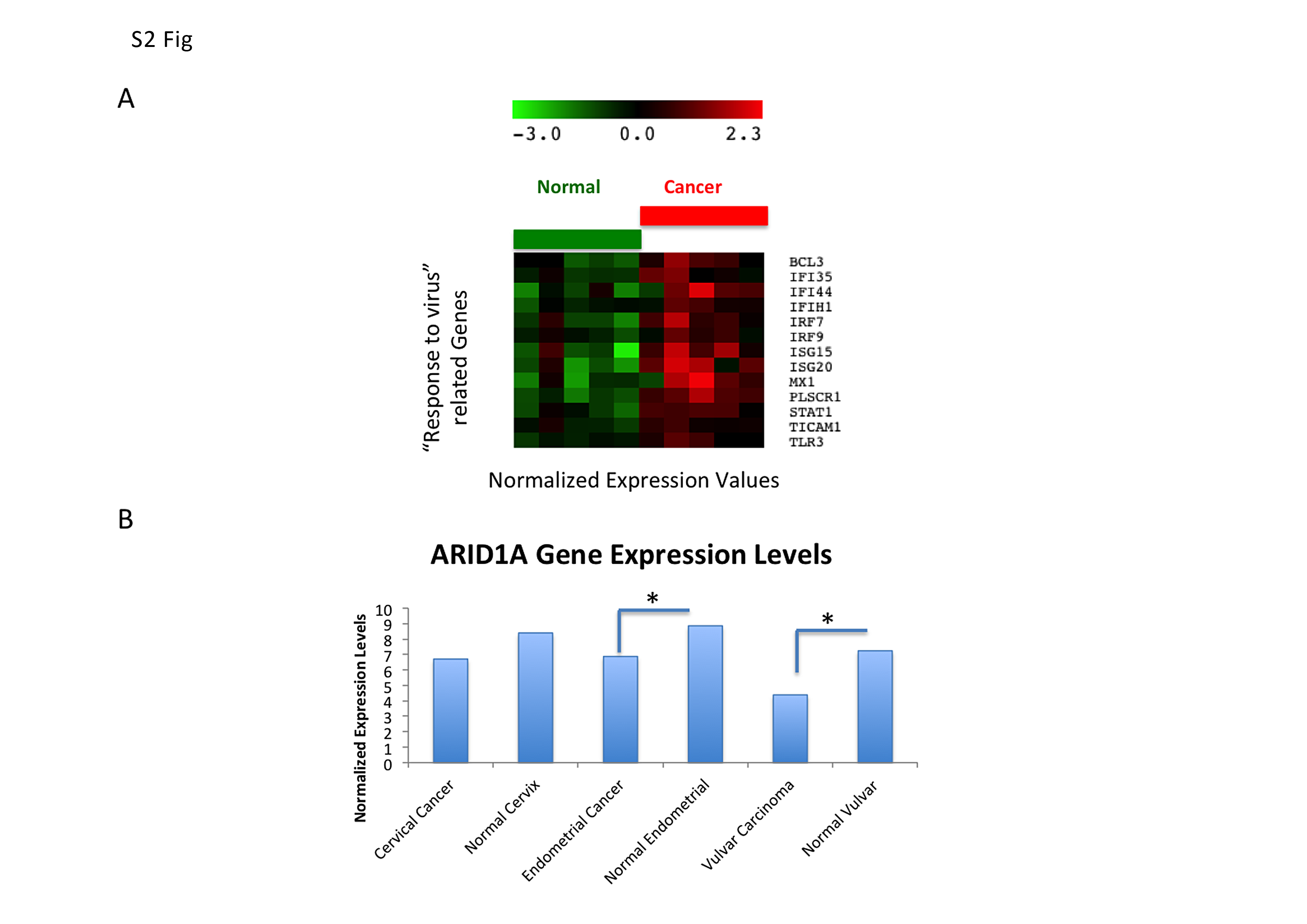

Supplement: S2 Fig — A. Heatmap of normalized cervical cancer samples showing differentially expressed genes involved in ‘response to virus’. B. Average ARID1A expression levels in the three gynecological cancers and their corresponding normal samples. Though ARID1A was found downregulated (p <0.05) in endometrial and vulvar cancer, vulvar cancer samples exhibited greater reduction (fold change = -1.7 in vulvar cancer vs -1.3 in endometrial cancer). For significant differences with p < 0.05, an asterisk (*) was used for annotation. (TIF) [file pone.0142229.s002.tif]

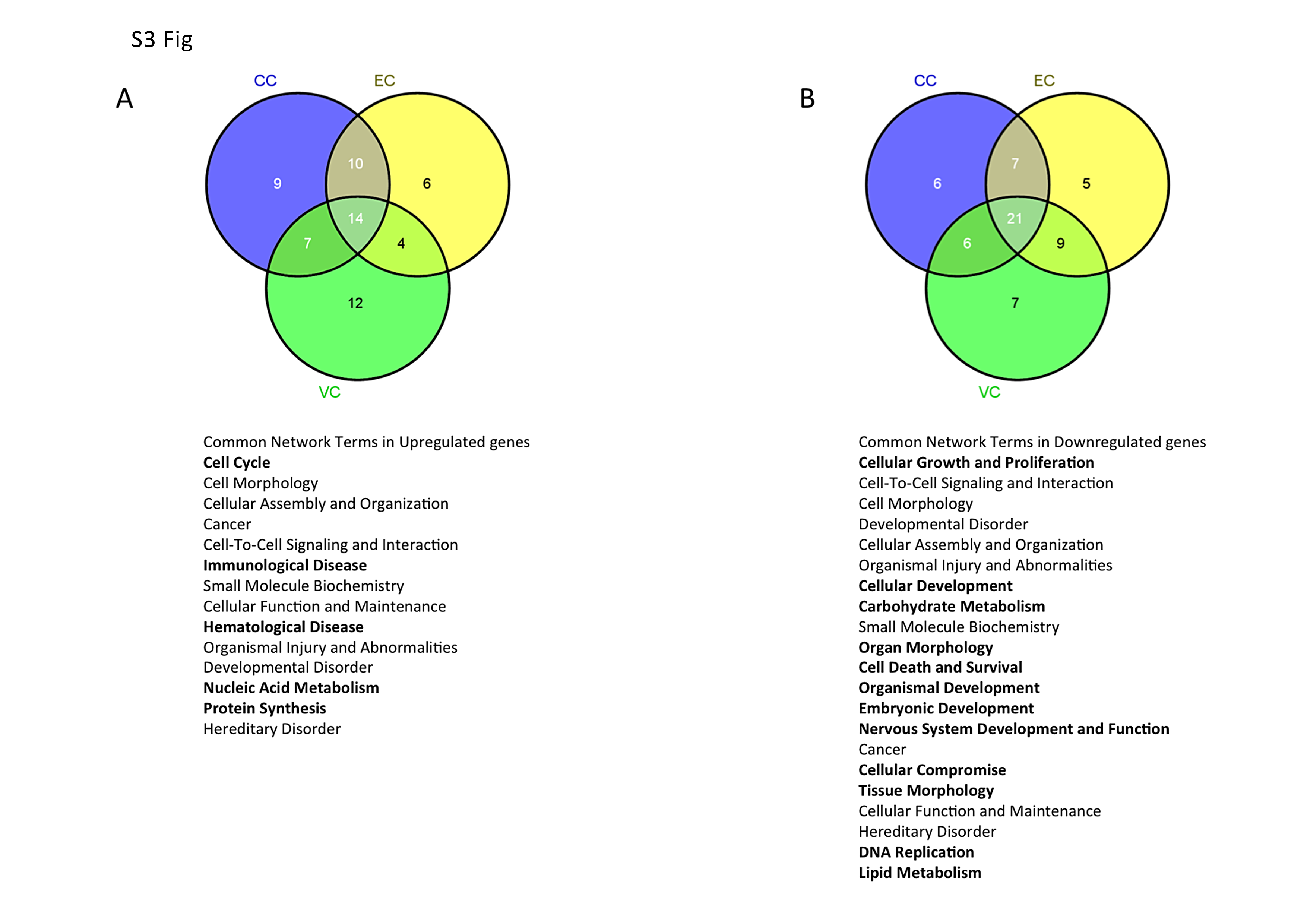

Supplement: S3 Fig — A. Venn diagram comparing the terms in network formation from IPA software in upregulated genes. B. Venn diagrams of downregulated genes in the three gynecological cancers of the study. Below are shown the common network terms in each comparison. The categories that are unique in upregulated and downregulated common network terms are shown in bold. (TIF) [file pone.0142229.s003.tif]

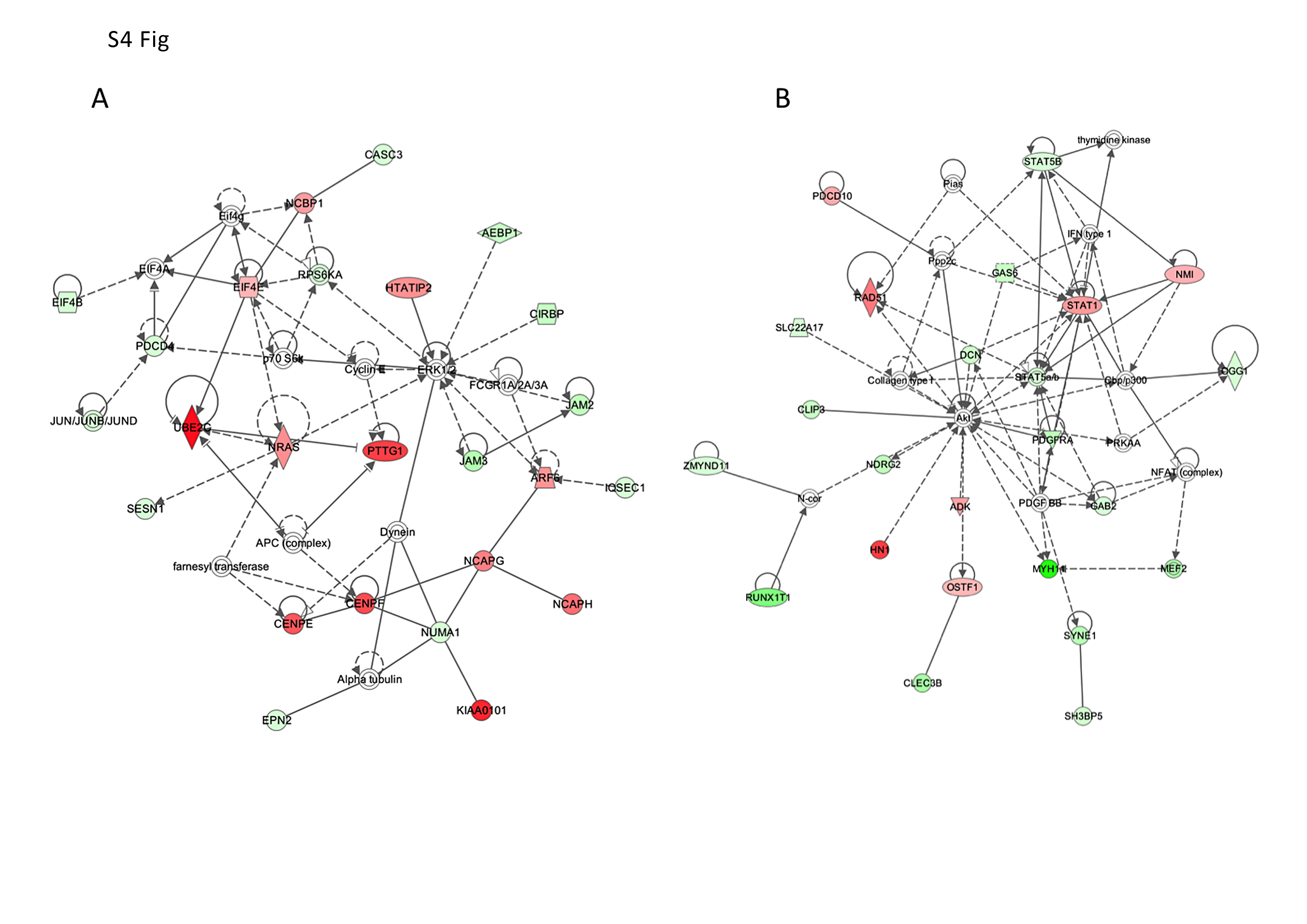

Supplement: S4 Fig — Networks formed with IPA using the common regulated genes from all gynecological cancers (193 genes). A. Cell cycle-related network. B. Cancer and Cell death and Survival-related networks were among the top three networks that exhibited the highest score. (TIF) [file pone.0142229.s004.tif]
