# Supplementary material for: Profiling of Discrete Gynecological Cancers Reveals Novel Transcriptional Modules and Common Features Shared by Other Cancer Types and Embryonic Stem Cells
Source: PLoS One. 2015 Nov 11;10(11):e0142229. doi: 10.1371/journal.pone.0142229 (PMC4641642; doi:10.1371/journal.pone.0142229)
Supplement: S1 Table — Clinicopathological features of the patients and normal controls of the study. Cancer cases were staged according to the 2009 FIGO staging guidelines [52]. (DOC) [file pone.0142229.s005.doc]

| **S1 Table.** Clinicopathological features of the 18 patients with gynecological cancer and of the 17 women of the control group. | | | | | | | |
| --- | --- | --- | --- | --- | --- | --- | --- |
| **No.** | **Code-M** | **Code-C** | **Age** | **Tissue type** | **Pathology** | **Grade** | **Stage** |
| **1** | A101 | Ce36 | 49 | Cervical cancer | Non-keratinizing invasive squamous cell carcinoma | 3 | Ib1 |
| **6** | A107 | Ce38 | 38 | Cervical cancer | Mucinous adenocarcinoma | 3 | Ib2 |
| **20** | D236 | MC35 | 68 | Cervical cancer | Squamous carcinoma | 2 | IIIb |
| **21** | D237 | MC4 | 55 | Cervical cancer | Squamous carcinoma | 2 | IIIb |
| **32** | D399 | MC1 | 41 | Cervical cancer | Squamous carcinoma | 0 | Ia1 |
| **2** | A102 | CeN11 | 52 | Cervical tissue control | Benign ovarian cyst |  |  |
| **16** | A140 | CeN12 | 45 | Cervical tissue control | Fibroid |  |  |
| **23** | D240 | MCN7 | 52 | Cervical tissue control | Benign ovarian cyst |  |  |
| **24** | D241 | MCN17 | 45 | Cervical tissue control | Fibroid |  |  |
| **35** | Xd242 | MCN18 | 64 | Cervical tissue control | Benign ovarian cyst |  |  |
| **7** | A109 | ME27 | 39 | Endometrial cancer | Endometrioid adenocarcinoma | 3 | IIIa |
| **11** | A124 | E12 | 69 | Endometrial cancer | Endometrioid adenocarcinoma | 2 | Ib |
| **25** | D243 | ME15 | 52 | Endometrial cancer | Mixed endometrioid adenocarcinoma and clear cell | 2 | Ib |
| **26** | D244 | ME16 | 65 | Endometrial cancer | Adenocarcinoma with squamous differentiation | 1 | Ia |
| **27** | D245 | ME20 | 52 | Endometrial cancer | Mixed endometrioid adenocarcinoma, clear cell and papillary serous | 3 | IV |
| **28** | D246 | ME19 | 42 | Endometrial cancer | Adenocarcinoma with squamous differentiation | 2 | Ib |
| **34** | T68 | E1 | 69 | Endometrial cancer | Mixed endometrioid adenocarcinoma and villoglandular | 3 | Ib |
| **3** | A104 | EN7 | 52 | Endometrial tissue control | Benign ovarian cyst |  |  |
| **8** | A110 | EN11 | 45 | Endometrial tissue control | Uterine myomas |  |  |
| **29** | D247 | MEN6 | 36 | Endometrial tissue control | Uterine myomas |  |  |
| **30** | D248 | MEN10 | 33 | Endometrial tissue control | Uterine myomas |  |  |
| **31** | D249 | MEN12 | 45 | Endometrial tissue control | Uterine myomas |  |  |
| **4** | A105 | V21 | 77 | Vulvar cancer | Keratinized squamous | 1 | II |
| **9** | A111 | V34 | 78 | Vulvar cancer | Keratinized squamous | 1 | II |
| **12** | A126 | V26 | 82 | Vulvar cancer | Keratinized squamous (recurrent) | 1 | IIIb |
| **13** | A127 | V32 | 79 | Vulvar cancer | Keratinized squamous | 3 | IIIb |
| **15** | A139 | V33 | 78 | Vulvar cancer | Keratinized squamous | 2 | Ib |
| **33** | D400 | MV17 | 70 | Vulvar cancer | Keratinized squamous | 2 | IVb |
| **5** | A106 | VN26 | 70 | Vulvar tissue control | Uterine prolapse |  |  |
| **10** | A112 | VN27 | 55 | Vulvar tissue control | Uterine prolapse |  |  |
| **14** | A128 | VN42 | 56 | Vulvar tissue control | Uterine prolapse |  |  |
| **17** | A870 | VN28 | 55 | Vulvar tissue control | Uterine prolapse |  |  |
| **18** | A871 | VN29 | 57 | Vulvar tissue control | Uterine prolapse |  |  |
| **19** | A872 | VN50 | 70 | Vulvar tissue control | Uterine prolapse |  |  |
| **22** | D239 | MVN3 | 56 | Vulvar tissue control | Uterine prolapse |  |  |
