# Supplementary material for: Profiling of Discrete Gynecological Cancers Reveals Novel Transcriptional Modules and Common Features Shared by Other Cancer Types and Embryonic Stem Cells
Source: PLoS One. 2015 Nov 11;10(11):e0142229. doi: 10.1371/journal.pone.0142229 (PMC4641642; doi:10.1371/journal.pone.0142229)
Supplement: S5 Table — List of GEO accession codes used for comparative analysis of the expression profile of cervical cancer samples with HeLa, A549, K562, HepG2 and normal brain cells. (DOC) [file pone.0142229.s009.doc]

| **Cell Line** | **Study GSE accession code** |
| --- | --- |
| HeLa | GSE23103 |
|  | GSE26868 |
|  | GSE37935 |
|  | GSE41827 |
|  | GSE56168 |
| A549 | GSE6013 |
|  | GSE14315 |
|  | GSE17307 |
|  | GSE32496 |
| K562 | GSE12056 |
|  | GSE14083 |
|  | GSE16085 |
|  | GSE20928 |
|  | GSE57470 |
| HepG2 | GSE15162 |
|  | GSE18269 |
|  | GSE25547 |
| Brain | GSE15209 |
|  | GSE28160 |
|  | GSE30563 |

**S3 Table**. List of studies representing different cell lines used for correlation analysis with the expression profile of cervical cancer samples.
